# Childhood-Onset Granulomatosis With Polyangiitis as a Palatal Defect: A Case Report

**DOI:** 10.7759/cureus.42855

**Published:** 2023-08-02

**Authors:** Brandon W Knopp, Jessica Baran, Robert Casey

**Affiliations:** 1 Endocrinology, Florida Atlantic University Charles E. Schmidt College of Medicine, Boca Raton, USA; 2 Emergency Medicine, Florida Atlantic University Charles E. Schmidt College of Medicine, Boca Raton, USA; 3 Pediatrics, Joe DiMaggio Children's Hospital, Hollywood, USA

**Keywords:** emergency department, autoimmunity, granulomatous vasculitis, palatal defect, pediatrics, eosinophilic granulomatosis polyangiitis

## Abstract

Granulomatosis with polyangiitis (GPA) is a necrotizing systemic vasculitis of small and medium-sized vessels with renal and sinopulmonary involvement. Its symptoms include chronic sinusitis, recurrent pneumonia, glomerulonephritis, constitutional symptoms, and skin manifestations with a typical onset in the fourth to sixth decade of life. We present a rare case of GPA in a 16-year-old female who presented with facial numbness and nasal regurgitation via a palatal defect. The patient reported a several-month history of recurrent epistaxis and chronic nasal congestion accompanied by several weeks of night sweats, lower right-sided facial numbness and pain, nasal regurgitation of food and liquids, and a 30-pound weight loss. A physical exam found a 3-cm defect on the right side of her palate. CT of the sinuses showed significant sinonasal destruction and petrous apicitis. GPA was confirmed via pathognomonic chest X-ray findings and biopsy results. The patient was treated with maxillary antrostomy and anterior ethmoidectomy and a follow-up was scheduled to address sequelae of the destructive sinopulmonary lesions. This case report highlights a unique presentation of GPA with an insidious development of autoimmune sinonasal destruction in an adolescent female. This presentation is rare and highlights the importance of considering autoimmune disease in cases of tissue destruction where the etiology is not apparent, even in patients at low risk for autoimmune conditions.

## Introduction

Granulomatosis with polyangiitis (GPA) is an autoimmune disease characterized by necrotizing systemic vasculitis of small and medium-sized vessels, including small arteries, arterioles, venules, and capillaries, with renal and sinopulmonary involvement [1,2]. Disease onset is typically seen in the fourth to sixth decade of life with symptoms including chronic sinusitis, recurrent pneumonia, glomerulonephritis, constitutional symptoms, and skin manifestations (ulcers, papules, vesicles) [3]. GPA is rarely observed in pediatric patients, with an incidence rate of 1.8 cases/million person-years as compared to the adult form with an incidence rate of 12.8 cases/million person-years [4]. Childhood-onset GPA (age <18 years) presents similarly to adult-onset GPA, albeit with some key differences. Childhood-onset GPA more commonly affects the ears, nose, and throat (ENT) system, and constitutional, renal, lower respiratory tract, musculoskeletal, and cutaneous involvement has been reported as well [5]. It is associated with more severe complications such as subglottic stenosis (five times greater risk in children) and nasal deformity (two times greater risk in children). This condition has high long-term morbidity characterized by frequent relapses and symptoms including leukopenia, neutropenia, and hypogammaglobulinemia [4,5,6]. The long-term prognosis of childhood-onset GPA is largely determined by the degree of renal involvement, with dialysis and renal transplantation being potential complications of early renal involvement [5,7]. However, there is scant data available regarding the frequency of renal involvement or other disease presentations in childhood-onset GPA.

The sparsity of information regarding childhood-onset GPA, in addition to the increased potential for severe complications, complicates its diagnosis as there is greater uncertainty in diagnostic criteria. Hence, it is imperative for clinicians to have a better understanding of the potential presentations of childhood-onset GPA and a higher index of clinical suspicion regarding GPA in undiagnosed pediatric patients. In this report, we present the case of a 16-year-old female diagnosed with GPA after presenting with nasal regurgitation and a right-sided palatal defect. The patient was initially suspected to have a cleft palate but was eventually diagnosed with GPA following the discovery of granulomatous sinopulmonary lesions pathognomonic for GPA.

## Case presentation

The patient was a 16-year-old female with a history of recurrent epistaxis, chronic nasal congestion, and recurrent otitis media who presented with several weeks of nasal regurgitation of food and liquids. A review of systems was significant for night sweats, a 30-pound weight loss over the past several weeks, headaches, blurred vision in the right eye, loss of smell, and lower right-sided facial numbness and pain. She reported odynophagia for the past three weeks and could only consume liquids through a straw due to nasal regurgitation. Her family history was significant for lupus on the paternal side and recurrent ear infections in her father and brother. She reported no oral ulcers, strawberry gingivitis, wheezing, cough, hoarseness, hemoptysis, pleuritic pain, arthralgias, hematuria, or dysuria.

**Figure 1:**
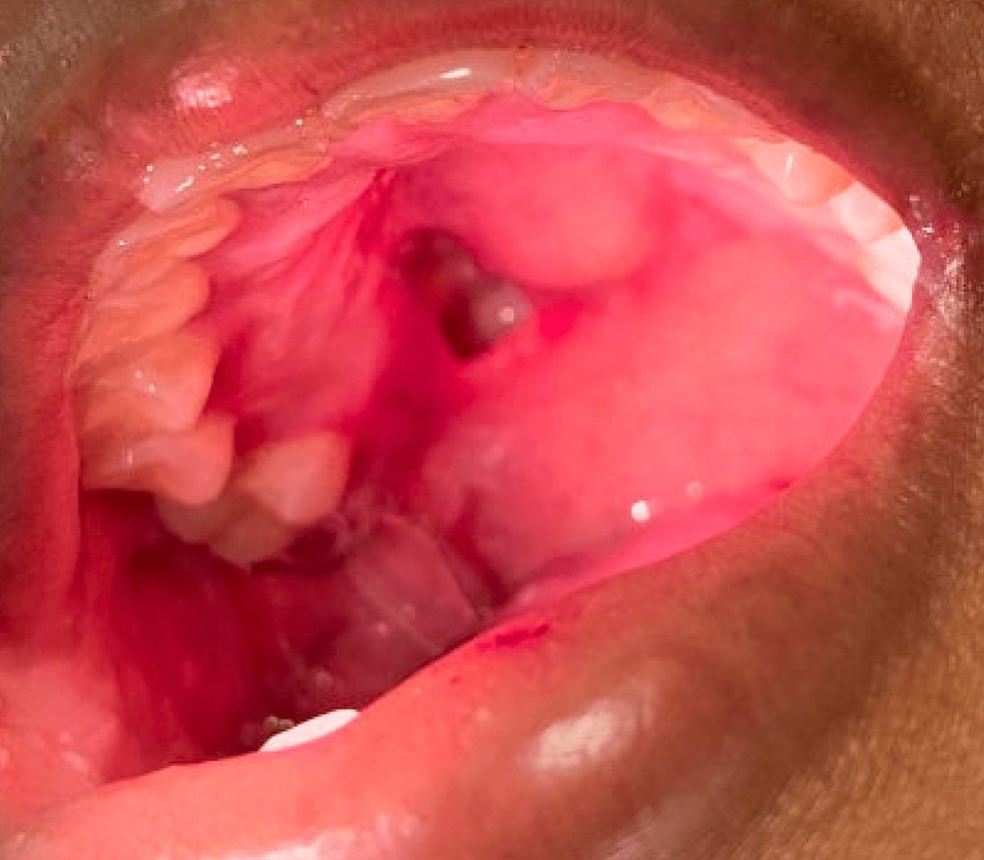
Right palatal defect

Vitals were largely unremarkable with an average temperature of 38.2 ^o^C and a max temperature of 39.1 ^o^C in the first 24 hours. A physical exam found an approximately 3-cm defect on the right side of the posterior hard palate and anterior soft palate with no surrounding erythema or ulcerative lesions (Figure 1).

She had right-sided facial tenderness and loss of sensation. Central tympanic membrane perforation (<15%) was present on the right side with tympanic membrane opacification and mucoid middle ear effusion superiorly. Extraocular movements were intact, pupils were equal and reactive to light, and there was no periorbital erythema or edema. Her left eye vision was 20/200 and her right eye vision was 20/400. She could not differentiate the color red (worse in the right eye) and had no pain with extraocular movements. Nares were congested with a foul odor and blood clots were present bilaterally. The bilateral parotid glands were edematous, firm, and markedly tender to palpitation. Cranial nerves II-VII were grossly intact with no facial asymmetry noted. Deep ulcerations were observed at the top of the umbilicus and left upper arm. Superficial ulcerations were observed along the labia majora and perineal region. Multiple nodules were palpated subcutaneously in the upper arms.

**Figure 2:**
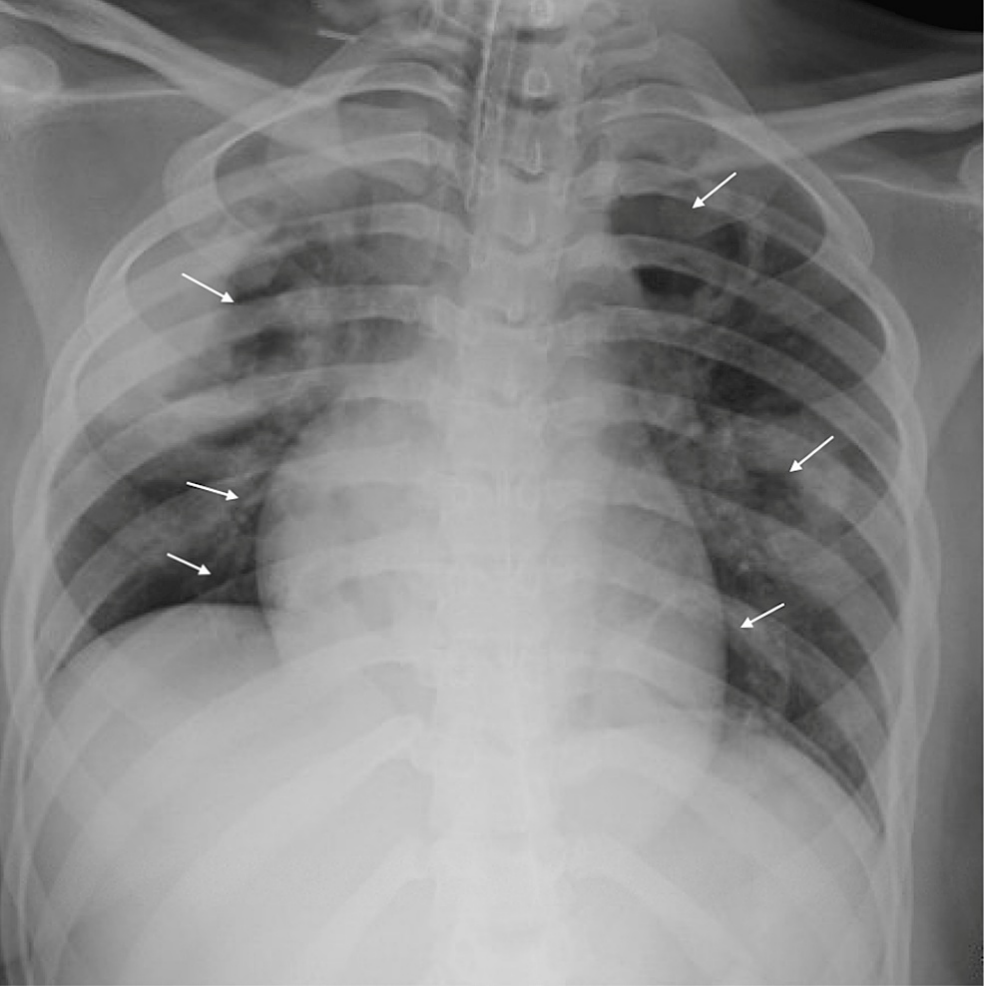
Chest X-ray with granulomatous lesions Arrows point to multiple granulomatous lesions

**Figure 3:**
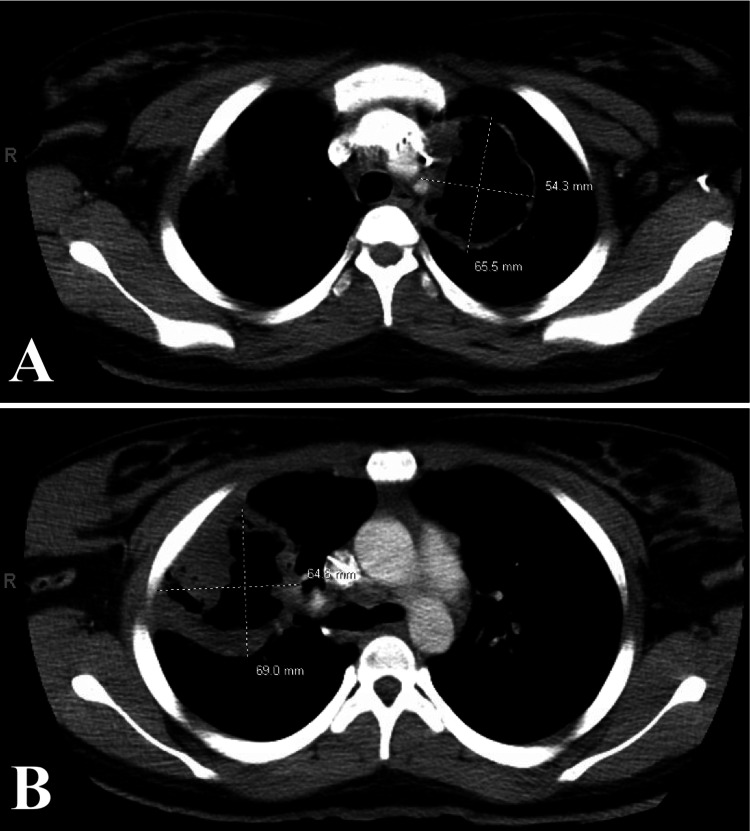
CT chest with cavitating lesions in the left (A) and right (B) lungs Cross-sectional cavity measurements displayed in A and B CT: computed tomography

**Figure 4:**
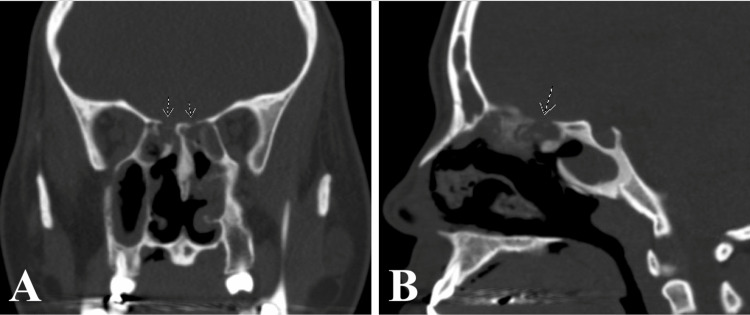
CT of the sinuses in AP (A) and lateral (B) views with evidence of diffuse tissue destruction Arrows point to destructive bone lesions CT: computed tomography; AP: anteroposterior

Bronchoscopy revealed diffuse airway bleeding with erythematous and friable mucosa in the bronchioles. Chest X-ray and follow-up chest CT scan showed cavitary opacities in the upper and lower lungs bilaterally, with the largest cavitary mass measuring 6.9 cm in diameter in the right upper lobe (Figures 2, 3). Significant sinonasal destruction and petrous apicitis were noted on a CT of the sinuses (Figure 4).

A right orbital abscess was noted and later found positive for Staphylococcus aureus and group B streptococcus, both with wide antibiotic susceptibility. Imaging also revealed extraocular muscle thinning and diffuse lesions indicative of bony destruction extending into the right superomedial orbit, nasal septum, posterior ethmoid sinus, right anterior sphenoid sinus, posterior hard palate, and right anterior soft palate. Lesions were predominantly on the right side. Nasal biopsies revealed diffuse necrotizing vasculitis with foci of granulomatous inflammation. Biopsies of the left arm and shoulder lesions showed leukocytoclastic vasculitis consistent with small- to medium-sized vessel vasculitis. GPA was confirmed based on pathognomonic X-ray chest lesions and the above-mentioned biopsy results. The patient also tested positive for cytoplasmic antineutrophil cytoplasmic antibody (C-ANCA). Notable laboratory results are presented in Table 1.

**Table 1:** Notable laboratory results

Variable	Results on admission	Results on discharge	Reference values
White blood cell count	20,100	14,600	4500–11,000/uL
Absolute neutrophil count	18,420	13,820	1820–7470/uL
Hemoglobin	9.4	9.1	11.4–15.4g/dL
Hematocrit	28.2	28.5	36.0–49.0%
Mean corpuscular volume	70.9	74.4	80–100fL
Platelet count	728	683	150,000–450,000/uL

Additional findings included the immunological markers summarized in Table 2 and a urinalysis significant for cloudy urine, 2+ leukocyte esterase, 11-20 white blood cells per high-powered field (HPF), 41-50 red blood cells per HPF, and 3+ blood. Renal ultrasound was unremarkable, the viral and bacterial panels were negative, and cultures of the sinuses, blood, and urine showed no growth after five days. Neutrophil oxidase burst assay showed normal neutrophil function.

**Table 2:** Flow cytometry

	Results on admission	Reference values
CD3%	46.33%	56.00%–84.00%
CD3 absolute (cells/uL)	460	1000–2200
CD4%	25.72%	31.00%–52.00%
CD4 T cell absolute (cells/uL)	255	530–1300
CD8%	19.61%	18.00%–35.00%
CD8 T cell absolute (cells/uL)	195	330–920
CD4/CD8 (ratio)	1.31	1.00–6.90
CD19% (B cells)	28.2%	6.0%–23.0%
CD19, absolute (B cells) (cells/uL)	280	110–570
CD56% (NK cells)	24.5%	3.0%–22.0%
CD56, absolute (NK cells) (cells/uL)	243	70–480

The patient was intubated for bronchoscopy with maxillary antrostomy and anterior ethmoidectomy with multiple biopsies taken. She remained intubated for a total of three days on minimal ventilator settings due to the presence of extensive sinopulmonary inflammation and necrosis. On admission, she had received ampicillin/sulbactam and three days of pulse steroids due to suspicions of GPA before biopsy confirmation. The patient was placed on rituximab, prednisolone, and prophylactic antibiotics. She was discharged on hospital day seven with instructions to regularly use saline irrigation and mupirocin rinses and gels to reduce sinus symptoms. Rituximab infusions were scheduled for every two weeks. The patient was counseled on her condition and informed of the findings. On ENT follow-up two weeks later, the patient reported improved vision, continued nasal regurgitation, and continued nose crusting. She was scheduled for a plastic surgery consultation to evaluate a closure of her palatal defect and for regular ENT follow-ups for long-term disease management.

## Discussion

This case report highlights a unique presentation of GPA characterized by the insidious development of autoimmune sinonasal destruction in an adolescent female. The patient presented with nasal regurgitation secondary to a hard palate defect, though further evaluation found widespread autoimmune destruction, most prominent in the sinonasal passages. This case was unique given the rarity of childhood-onset GPA and the atypical presentation with nasal regurgitation. On further evaluation, the patient reported widespread symptoms including night sweats, weight loss, chronic nasal congestion, recurrent otitis media, recurrent nosebleeds, facial numbness, blurred vision, odynophagia, and skin nodules. These widespread symptoms, some not initially reported, were more suggestive of a systemic process and prompted further diagnostic workup. However, the absence of lower respiratory and renal symptoms complicated the diagnosis as the presenting symptoms were largely non-specific. The palatal defect was initially suspected to be due to a submucous cleft palate defect with mucous membrane idiopathic destruction. However, the extent of additional symptoms made a systemic process more likely.

Infectious and autoimmune etiologies were investigated, with orbital and pulmonary abscesses found on imaging, although sinus, blood, and urine cultures were all negative. A high degree of evidence was found to support an autoimmune etiology with elevated white blood cells, an elevated absolute neutrophil count, a high percentage of CD19+ cells (B cells), a high percentage of CD56+ cells (NK cells), and evidence of necrotizing vasculitis with granulomatous inflammation on multiple biopsies. The pathognomonic cavitating lesions seen in the lungs bilaterally on chest X-ray and positive C-ANCA screening confirmed the diagnosis of GPA. Childhood-onset GPA typically presents similarly to adult-onset GPA with the long-term prognosis largely determined by the degree of renal involvement. Cases with greater renal involvement have a greater risk of developing end-stage renal disease, potentially leading to dialysis or transplantation [7]. Other aspects of childhood-onset GPA symptoms and prognosis have yet to be well-described, due to the scarcity of GPA cases in children and the subsequent lack of research into childhood-onset GPA [5].

Data on childhood-onset GPA in the literature is mostly confined to case series, case reports, and small cohort studies. Similar to other reported pediatric GPA cases, our patient had autoimmune sinopulmonary involvement at presentation with signs of multisystem disease [5]. Unlike many other childhood-onset GPA cases, this patient reported no pulmonary or renal symptoms, and such involvement was only discovered later following chest X-ray and urinalysis, respectively [1,2,8,9]. This is the first reported case of a pediatric patient with GPA presenting with primarily sinonasal involvement and no reported pulmonary or renal symptoms.

## Conclusions

This report described a unique presentation of childhood-onset GPA. Our patient’s presenting complaint was a palatal defect, with constitutional signs of systemic disease reported by the patient only later. This case demonstrates one potential presentation of childhood-onset GPA and highlights the importance of considering GPA in pediatric patients with destructive sinonasal changes, even in the absence of symptomatic pulmonary and renal disease.
